# 2021 SAEM Consensus Conference Proceedings: Research Priorities for Implementing Emergency Department Screening for Social Risks and Needs

**DOI:** 10.5811/westjem.2022.10.57368

**Published:** 2023-02-24

**Authors:** Mackensie Yore, Callan Elswick Fockele, Herbert C. Duber, Kelly M. Doran, Richelle J. Cooper, Michelle P. Lin, Steffani Campbell, Vidya Eswaran, Betty Chang, Haeyeon Hong, Kessiena Gbenedio, Kimberly A. Stanford, Nicholas Gavin

**Affiliations:** * VA Los Angeles and UCLA National Clinician Scholars Program, VA Greater Los Angeles Healthcare System HSR&D Center of Innovation, Los Angeles, California; † University of Washington, Department of Emergency Medicine, Seattle, Washington; ‡ NYU Grossman School of Medicine, Departments of Emergency Medicine and Population Health, New York, New York; § UCLA David Geffen School of Medicine, UCLA Department of Emergency Medicine, Los Angeles, California; ¶ Baylor College of Medicine, Department of Emergency Medicine and Section of Health Services Research, Department of Medicine, Houston, Texas; || Stanford University, Department of Emergency Medicine, Stanford, California; # UCSF Fresno, Department of Emergency Medicine, Fresno, California; ** Columbia University, Department of Emergency Medicine, New York, New York; †† Boston Medical Center, Department of Emergency Medicine, Boston, Massachusetts; ‡‡ University of Chicago, Section of Emergency Medicine, Chicago, Illinois; §§ Mount Sinai Icahn School of Medicine, Department of Emergency Medicine, New York, New York

## Abstract

**Introduction:**

Despite literature on a variety of social risks and needs screening interventions in emergency department (ED) settings, there is no universally accepted or evidence-based process for conducting such interventions. Many factors hamper or promote implementation of social risks and needs screening in the ED, but the relative impact of these factors and how best to mitigate/leverage them is unknown.

**Methods:**

Drawing on an extensive literature review, expert assessment, and feedback from participants in the 2021 Society for Academic Emergency Medicine Consensus Conference through moderated discussions and follow-up surveys, we identified research gaps and rated research priorities for implementing screening for social risks and needs in the ED. We identified three main knowledge gaps: 1) screening implementation mechanics; 2) outreach and engagement with communities; and 3) addressing barriers and leveraging facilitators to screening. Within these gaps, we identified 12 high-priority research questions as well as research methods for future studies.

**Results:**

Consensus Conference participants broadly agreed that social risks and needs screening is generally acceptable to patients and clinicians and feasible in an ED setting. Our literature review and conference discussion identified several research gaps in the specific mechanics of screening implementation, including screening and referral team composition, workflow, and use of technology. Discussions also highlighted a need for more collaboration with stakeholders in screening design and implementation. Additionally, discussions identified the need for studies using adaptive designs or hybrid effectiveness-implementation models to test multiple strategies for implementation and sustainability.

**Conclusion:**

Through a robust consensus process we developed an actionable research agenda for implementing social risks and needs screening in EDs. Future work in this area should use implementation science frameworks and research best practices to further develop and refine ED screening for social risks and needs and to address barriers as well as leverage facilitators to such screening.

## INTRODUCTION

Adverse social determinants of health, which encompass a host of socioeconomic and behavioral factors, are primary drivers of illness and injury.^1^ The set of adverse social factors linked to an individual’s poor health is referred to as their “social risk,” while their expressed priorities and desires for assistance addressing their social risks are collectively referred to as their “social need.”^2^,^3^

The emergency department (ED) provides a unique and important setting for social risks and needs screening and intervention to provide higher value care.^4^ Social risks and needs such as housing instability, food insecurity, lack of employment, substance use, and transportation barriers are prevalent in the ED patient population.^5^–^9^ Furthermore, approximately a quarter of adults in the United States lack a usual source of medical care.^10^ This group, particularly those uninsured or enrolled in Medicaid, often relies on the ED when health issues arise,^11^ highlighting a need for the ED to provide screening and resources that many patients are unable to access elsewhere. However, many factors affect the implementation of social risks and needs screening in the ED, including screening tool characteristics and deployment, stakeholder perspectives on screening, characteristics of the clinical, reimbursement, and regulatory environments, and the selected implementation strategies.^12^ The impact of these factors on screening implementation and uptake is inadequately characterized. A better understanding of the components and steps involved in implementing efficient and impactful ED-based social risks and needs screening programs could facilitate the uptake of this important tool for addressing the social determinants of health.

To spur research on ED-based social risks and needs screening, the 2021 Society for Academic Emergency Medicine (SAEM) Consensus Conference, “From Bedside to Policy: Advancing Social Emergency Medicine and Population Health through Research, Collaboration and Education” (“Consensus Conference”) developed a research agenda based on literature gaps, expert opinion, and stakeholder feedback comprising the following: 1) instruments for social risks and needs screening in the ED; 2) implementation of social risks and needs screening in the ED; and 3) interventions for patients with identified social risks and needs in the ED. Our goal in this article, the second of three manuscripts, was to describe consensus, process-derived research gaps and priorities related to *implementation* of social risks and needs screening in the ED setting.

## METHODS

**Figure 1:**
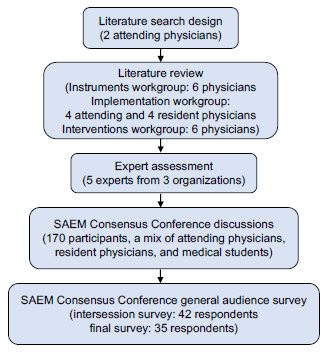
2021 Society of Academic Emergency Medicine (SAEM) Consensus Conference process for identifying research gaps and priorities for implementation of emergency department-based social risks and needs screening.

We identified research gaps and priorities for ED-based social risks and needs screening instruments, implementation, and interventions through a consensus-based approach, drawing on an extensive literature review, expert consultation, and feedback from Consensus Conference participants during moderated discussions and follow-up surveys (Figure 1).

**Population Health Research Capsule**

What do we already know about this issue?

*The ED is an important setting for social risks/needs screening and intervention, yet factors affecting screening implementation are poorly characterized*.

What was the research question?

*What are the research gaps and priorities related to implementation of social risks/needs screening in the ED setting?*

What was the major finding of the study? Major comparison with p-value and confidence interval

*In a consensus process, we developed and ranked 12 research questions to address three social risks/ needs screening implementation knowledge gaps*.

How does this improve population health?

*We highlight research needed on the design, structure, and operationalization of ED social risks/needs screening to increase program sustainability and patient benefit*.

### Literature Review

**Figure 2:**
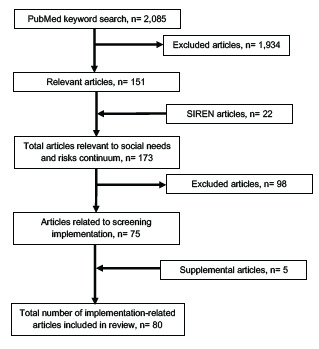
Flow diagram of literature review search results. *SIREN*, Social Interventions Research & Evaluation Network.

A literature review on social risks and needs screening in the ED, adapted from methods used by Malecha et al^5^ and in consultation with a social sciences librarian, identified 2,085 articles covering screening tools, implementation, and/or interventions (Figure 2). Based on relevance of titles and abstract content, we selected 151 articles for detailed review. We found another 188 articles using the search term “emergency” in the Social Interventions Research & Evaluation Network (SIREN) Evidence and Resource Library^13^ and selected 22 for detailed review. Both searches were conducted in December 2020. Of the 173 articles identified for detailed review, 75 addressed implementation of ED screening, focusing on screening format and workflow, team structure, and barriers and facilitators to screening implementation.

Finally, five additional articles from bibliographic references of the reviewed manuscripts were added to the literature review, based on their pertinence to ED screening implementation. A team of four attending and four resident physicians, all in Emergency Medicine, reviewed the 80 articles and extracted details into an Excel for Mac, version 16.52 (Microsoft Corp, Redmond, WA) database with information on study objective, design, outcomes, results, limitations, and quality. Our workgroup analyzed the extracted data and source manuscripts with the primary goals to identify research gaps and to subsequently draft research priorities.

### Engagement and Feedback

We shared these draft research priorities with a panel of experts drawn from three organizations: the Office of the Assistant Secretary for Planning and Evaluation, a health policy-focused government agency^14^; Health Leads, a nonprofit organization connecting communities to social resources^15^; and SIREN, a program at the University of California San Francisco that researches healthcare sector strategies to address social conditions.^13^ We integrated feedback from these experts into a pre-reading document shared with Consensus Conference participants.

The SAEM Consensus Conference was held virtually using Zoom sessions (Zoom Video Communications, San Jose, CA) on April 13 and 27, 2021. The first session included a moderated discussion of methods, research gaps, and preliminary research priorities that incorporated expert feedback regarding the implementation of social risks and needs screening in the ED. After the first session, an intersession survey gathered feedback from the Consensus Conference participants, and this feedback was integrated into a revised set of research priorities. In the second session, moderated discussion further refined the priorities and ratings and resulted in a revised list of research priorities. In a final survey after the second session, participants ranked research priorities based on their perceived importance for future research and the SMART (specific, measurable, attainable, relevant, and time-based) criteria. Priorities were ranked using the following formula:


3 x (# of 1st choice votes)+2 x (# of 2nd choice votes)+1 x (# 3rd choice votes)=Total Score

We categorized research priorities as high, medium, or low priority based on relative score (top ⅓, middle ⅓, lowest ⅓, respectively). Below, we present the research priorities pertaining to implementation of social risks and needs screening, grouped by thematic gaps identified during the literature review.

## RESULTS AND DISCUSSION

Of the 80 articles reviewed, 10 were controlled clinical trials, including eight randomized controlled clinical trials, and five were prospective observational studies. Following the first moderated discussion, 31/32 survey respondents (96.8%) found that no additional priorities should be added to the research question list, and 28/32 (87.5%) recommended that no priorities be removed. Following the second Consensus Conference moderated discussion, 35 respondents completed the second survey, generating the final ranked list of research gaps and priorities, summarized in the Table and discussed in detail below.

### Gap 1: Screening Implementation Mechanics

Our literature review and Consensus Conference discussion identified several research gaps in the specific mechanics of screening implementation, including screening and referral team composition, workflow, and use of technology. Literature on social risks and needs screening describes the feasibility of, and potential concerns with, several team structures and workflows, including screening questions asked by ED staff (eg, registration clerk, nurse, social worker),^16^–^20^ completed independently by patients,^16^,^18^,^21^–^27^ or asked by external personnel (eg, patient navigator).^25^,^26^,^28^,^29^

Social risks and needs screening questions may be embedded in the electronic health record (EHR) and asked by ED staff in series with more conventional questions (eg, contact information, medical history, current symptoms).^30^ While this approach may integrate with the existing workflow and make use of staff already interacting with the patient, there may be a tendency by staff to rush or skip some questions given time constraints and the large volume of EHR prompts.^23^

Screenings completed independently by the patient often use electronic platforms such as tablets, kiosks, or chatbots.^16^,^21^,^23^–^26^,^31^,^32^ Such patient-facing, technology-based platforms can improve disclosure of risks/needs compared with face-to-face screening,^18^,^21^,^27^,^33^ especially in the ED waiting room and other spaces with limited privacy.^2^,^18^,^34^ Because these platforms do not require continuous staff time, screening can be more comprehensive, and patients have more autonomy over which questions to answer. Electronic screening can also automate referrals.^28^ Patient acceptance of self-facilitated, technology-based screening depends on patient age and screening topic; use of digital technologies is near-ubiquitous among adolescents,^34^ and most adolescents prefer technology-based screening for most social risk and need topics.

In 2000, increasing age was associated with lower acceptability of technology-assisted screening^35^; further studies could determine whether this sentiment persists and identify barriers to overcoming technological barriers among older adults. Another research gap is how technology might increase or impede screening accessibility for patients with vision or hearing impairments, limited English proficiency, and/or low health literacy. Furthermore, there is an opportunity for such research to include partnerships with patients in the co-design of accessible screening tools.

Several studies describe screening programs led by non-clinical staff and volunteers who can facilitate both screening and navigation to resources for identified needs (“patient navigator” model).^36^–^39^ Programs that specifically employ peer navigators and community health workers can incorporate community perspectives to better design screening programs, increasing patient comfort with disclosing needs, and empowering members of the community with new skills and opportunities.^40^ As with patient-completed questionnaires, screening not embedded within the EHR may lack EHR integration, and whether and how this information might be useful to clinicians and tracked over time is unstudied.

Consensus Conference participants broadly agreed that social risks and needs screening is generally acceptable to patients and clinicians and feasible in an ED setting. They therefore advocated that future research focus more on using best practices from quality improvement and implementation science to select and customize screening models to meet the needs of a local context, maximize the value of screening to patients and clinicians, and enable long-term sustainability of screening programs. For those new to quality improvement and implementation science, these practices may include using qualitative and quantitative methods to understand contextual factors and stakeholder perspectives, constructing testable theoretic and system models, and characterizing barriers and facilitators to initiating, scaling up, and sustaining screening.^41^–^43^ Additionally, researchers could plan experiments using one of many implementation research designs to evaluate screening deployment strategies through a combination of process and outcome metrics.^44^–^46^

Reflecting on the various models for screening, Consensus Conference participants expressed concern that screenings facilitated by overextended clinicians or nursing staff would be unsustainable regardless of buy-in and recommended research evaluating the screening by non-clinical staff (eg, peer navigators or college students) and/or training existing team members with nonclinical roles (eg, registration staff). Participants suggested clinicians would appreciate access to screening *results* even if they are less interested in doing the screening themselves. Participants recognized that many EDs have generally relied on social workers to address social needs of high-risk patients identified by clinicians and recommended that social workers be involved in the design and implementation of screening programs. Regardless of the screening model chosen, participants said it was essential for ED staff initiating or facilitating screening to understand and convey to patients the importance and utility of screening and demonstrate empathy throughout the process – an approach that may require additional training.

Research Priorities:

1. When should the screening be completed during the ED course? Where/how should it be done (eg, triage desk, registration, or alone in a treatment room; technology-assisted)? Where and with whom are the results of screening discussed?
2. What is the ideal team structure and skill-mix of personnel for supporting screening in the ED? How might community health workers, trained peers, and/or health system navigators be incorporated into the screening process?
3. What combination of interpersonal engagement and technology (eg, chatbots, kiosks, and EHR alerts and algorithms) in the screening process optimizes patient comfort disclosing their needs, maximizes efficiency, and facilitates successful referrals to community resources?
4. What is the comparative effectiveness of conducting a brief screening (eg, 1–2 items) for social risks/needs and then more detailed questions for those with potential risks/needs identified in the general screener versus starting with a more comprehensive screening for multiple discrete social risks/needs?
5. What is the comparative effectiveness and feasibility of strategies where interventions are triggered by positive social risks/needs screening versus universal offers of social needs assistance to ED patients?
6. What is the role of universal screening versus targeting certain patient groups (eg, patients with frequent ED visits)?

### Gap 2: Outreach and Community Engagement

The literature includes numerous examples of engagement between social risks and needs screening programs and external agencies, including community-based organizations (CBOs) and referral agencies, especially for linking patients with resources.^23^,^28^,^36^,^47^–^50^ Relationships with referral agencies and CBOs have so far been useful for refining screening tools,^23^ evaluating referral success,^49^ and sharing patients’ experiences.^50^ However, we found no studies that directly involved patients or CBOs in the *design* of ED screening processes.

During the Consensus Conference, participants discussed community outreach and engagement to 1) enhance bidirectional communication with referral agencies, and 2) make screening processes more patient-centered. Participants thought community partners could help tailor screening processes to particular settings (eg, rural areas, language minorities) and advise on the timeline and manner of screening. Furthermore, it was thought that involving referral agencies in program design could help these agencies better anticipate increased demand following screening implementation and help tailor the screening process to better match agencies’ purpose and capacity.

While some Consensus Conference participants advocated for a community-based participatory research approach to developing and implementing ED social risks and needs screening, we found no studies using this approach. Through such an approach, representatives from socially vulnerable communities could lead design of screening interventions centered on patients’ priorities; gather screening information (eg, through a community health worker approach); recommend resources that are most useful and referral agencies that are most trusted among the community; review and contextualize aggregate results (eg, trends in screening, numbers and types of referrals to various kinds of resources with community partners); and help evaluate and improve the program.^51^,^52^

Research Priorities:

1. How can EDs work effectively with and leverage existing expertise and resources of community organizations to optimize ED screening for social risks/needs?
2. How does the effectiveness of a given ED-based social risk/needs screening intervention vary across settings (ie, urban vs rural, academic vs community, and across multiple sites in general)? How can implementation of screening for social risks/needs be tailored based on setting to maximize effectiveness?

### Gap 3: Barriers and Facilitators to Screening

Our working group identified patient, personnel, system, and societal barriers to implementation of ED social risks and needs screening. Our literature review identified barriers and strategies to overcome these barriers and demonstrated research gaps that were further discussed and prioritized by Consensus Conference participants.

#### Patient-Related Barriers to Emergency Department Social Risks and Needs Screening

A variety of patient-related barriers to ED social risks and needs screening have been reported. Patient condition (eg, high-acuity illness, impairment) during the ED visit may limit screening of certain patients.^53^ Among patients able to be screened, those in hallway beds or other open areas may feel uncomfortable sharing screening information aloud.^54^ Others may be concerned about sharing information with unknown or untrusted referral organizations^54^ or triggering a report to Child Protective Services by disclosing certain risks (eg, intimate partner violence [IPV]).^55^ Furthermore, patients may decline screening due to disinterest in receiving resources.^56^ Factors that may facilitate screening in the ED include caring and empathetic interactions with screening staff, ability to immediately address identified needs,^55^ reassurance that screening will not delay care, assistance with screening technology, and observing that other patients are also screened.^16^ We found no studies that attempt to show the effect of addressing these barriers and facilitators on completion of screening, willingness to disclose risks and needs, or on accessing resources.

Consensus Conference participants described the lack of an ongoing patient-clinician relationship as a unique challenge for ED social risks and needs screening, highlighting a need for research to address which screening team structure (eg, clinical staff, peer navigators) is best for building trust to enable disclosure of social risks and needs and enable linkages to desired resources.

#### Personnel-Related Barriers to Emergency Department Social Risks and Needs Screening

Clinicians generally understand that social risks impact health,^4^ and most studies show clinical staff supporting the idea of screening 2,54,57 with greater support among physicians than nurses.^31^ Furthermore, attitudes toward screening can improve following implementation of screening programs.^58^ Clinical staff have also expressed reservations about screening, including a belief that screening is beyond their scope of practice,^59^,^60^ fear of offending patients,^28^,^55^,^59^,^61^,^62^ perceived or real lack of resources to address needs,^55^,^62^,^63^ and concern about disclosure increasing risk such as with IPV.^59^ In the case of IPV screening, however, evidence shows patient acceptability^23^,^53^,^54^ and satisfaction^64^ along with a single study finding no risk of violence with disclosure.^65^ Literature suggest several factors that may increase staff support for screening, including leveraging technology during screening^60^; selecting nurse champions to help direct implementation^16^; using a team approach to screening^60^; and ongoing staff engagement and feedback.^16^ Incentives for completing screening and disciplinary action for not screening have yielded mixed success,^57^,^66^ and staff-centered educational interventions alone to improve screening completion have shown limited efficacy.^21^,^63^

Consensus Conference participants noted that preparation for screening implementation often centered on training facilitators in content (eg, domestic violence, human trafficking), while insufficiently addressing critical system aspects such as funding, time, space, community engagement, and communication with referral agencies.

#### Systems-Related Barriers to Emergency Department Social Risks and Needs Screening

Our literature review identified multiple systems-level barriers to implementing social risk screening in the ED, including time constraints^2^,^55^,^61^,^62^,^67^,^68^; lack of established processes for addressing abuse^16^,^67^,^69^; and concern that screening may shift important ED resources away from acute care, lengthen ED stays, increase unreimbursed costs, and/or not be connected with appropriate interventions.^70^ Furthermore, while technology has the potential to make screening more efficient, certain “low-lift” technology strategies such as EHR alerts have not appreciably improved screening completion.^17^ Overall department culture and philosophy may also oppose social risk screening and challenge implementation.^67^

Consensus Conference participants noted that both rigorous quality improvement and implementation science begin with identifying local barriers to and facilitators for program success. Some participants recommended specific implementation frameworks, such as Exploration, Preparation, Implementation, and Sustainment^71^ and the Consolidated Frameworks for Implementation Research,^12^ as well as tools such as an Ishikawa diagram to identify factors within the local context contributing to efficient and accurate completion of screening and referral.^72^

#### Societal Barriers and the Payment/Policy Landscape

As insurance companies increasingly support value-based care, interest in addressing social determinants of health outside the hospital may increase. A current research gap is how payers and health systems can collaboratively address social risks and how to fairly attribute and compensate credit for successful interventions. We found no published literature evaluating the return on investment or cost-effectiveness for social risks screening in the ED, or on how incentives or mandates affect screening uptake. Participants identified incentives and regulation as critical to widespread implementation and called for rigorous studies (eg, multisite randomized control trials) demonstrating the ability for ED screening to ascertain and address patient social needs in order to justify these incentives and regulations.

Research Priorities:

1. What are patient-, clinician-, and systems-level barriers to social risks/needs screening in the ED? What strategies can be used to address the barriers to screening for social risks/needs in the ED? Do patient and clinician acceptability and accurate completion of screening improve when these barriers are addressed?
2. What is the “return on investment” for social risks/needs screening in the ED, considering broadly defined “returns” as well as costs (including time and resources) in the ED?
3. What strategies should be used to screen for social risks/needs among patients with psychiatric or high-acuity presentations? Non-English-speaking patients? Undocumented patients?
4. What factors of the payment and policy landscape (eg, mandates and funding) encourage/incentivize or discourage EDs from implementing social risk/need screening?

### Types of Studies Needed

Consensus Conference discussions identified the need for studies using adaptive designs or hybrid effectiveness-implementation models to test multiple strategies for implementation and sustainability, in part to justify large-scale funding to make screening routine. Mixed-methods studies were also encouraged to show not just feasibility but how and why screening works and how these interventions can be sustained.

## LIMITATIONS

This paper describes the series of activities leading to development of a research agenda on implementation of ED-based screening for social risks and needs as well as the research agenda itself. Although an extensive literature review was conducted at the beginning of this process, it was not designed as or intended to be a comprehensive systematic review. There is potential for omission of published or unpublished studies that might pertain to some of the research questions ultimately proposed. Furthermore, evidence was examined for quality, but no formal scoring with risk-of-bias tools was performed, as the goal was not to perform a systematic review but rather a focused, structured literature review to inform the consensus process. Another potential limitation is that the opinions and relative prioritization of research questions by the Consensus Conference participants could differ from opinions held by practitioners in the field more broadly.

## CONCLUSION

This paper presents research gaps and priorities in implementing ED social risks and needs screening identified using an iterative, consensus-based approach involving an extensive literature review, expert assessment, and feedback from participants in the 2021 SAEM Consensus Conference. While there is much to learn about the efficiency and efficacy of different ED-based social risks and needs screening modalities, literature to date has shown that screenings are acceptable to patients and lead to their engagement with interventions.^26^,^38^,^73^ We highlight a need for more collaboration with various stakeholders in screening design and implementation. This engagement should be paired with rigorous evaluation of screening implementation processes to identify best practices, particularly for patients from diverse groups, ensuring that all patients receive evidence-based interventions to improve social risk and health outcomes.

## Figures and Tables

**Table:** Final ranked research priorities pertaining to implementation of social risks and needs screening in the emergency department. Total points are weighted (3 points for priority 1 vote, 2 points for priority 2 vote, and 1 point for priority 3 vote).

Question	Priority			Total points	Priority category	Gap addressed*
	1	2	3			
How can EDs work effectively with and leverage existing expertise and resources of community organizations to optimize ED screening for social risk/needs?	10	3	4	40	High	OCE
What combination of interpersonal engagement and technology (eg, chatbots, kiosks, and EHR alerts and algorithms) in the screening process optimizes patient comfort disclosing their needs, maximizes efficiency, and facilitates successful referrals to community resources?	3	11	1	32	High	SIM
When should the screening be completed during the ED course? Where/how should it be done (eg, triage desk, registration, or alone in a treatment room; technology-assisted)? Where and with whom are results of screening discussed?	7	3	4	31	High	SIM
What are patient-, clinician-, and systems-level barriers to social risk/need screening in the ED? What strategies can be used to address the barriers to screening for social risk/needs in the ED? Do patient and clinician acceptability and accurate completion of screening improve when these barriers are addressed?	3	7	2	25	High	BFS
What is the ideal team structure and skill-mix of personnel for supporting screening in the ED? How might community health workers, trained peers, and/or health system navigators be incorporated into the screening process?	2	2	4	14	Medium	SIM
What is the comparative effectiveness of conducting a brief screening (eg, 1–2 items) for social risk/needs and then more detailed questions for those with potential risks/needs identified in the general screener versus starting with a more comprehensive screening for multiple discrete social risk/needs?	3	1	2	13	Medium	SIM
What is the “return on investment” for social risk/need screening in the ED, considering broadly defined “returns” as well as costs (including time and resources) in the ED?	0	3	7	13	Medium	BFS
How does the effectiveness of a given ED-based, social risk/need screening intervention vary across settings (ie, urban vs rural, academic vs community, and across multiple sites in general)? How can implementation of screening for social risk/needs be tailored based on setting to maximize effectiveness?	4	0	0	12	Medium	OCE
What strategies should be used to screen for social risk/needs among patients with psychiatric or high acuity presentations? Non-English-speaking patients? Undocumented patients?	0	3	5	11	Low	BFS
What is the comparative effectiveness and feasibility of strategies where interventions are triggered by positive social risk/need screening versus universal offers of social needs assistance to ED patients?	2	0	2	8	Low	SIM
What is the role of universal screening vs targeting certain patient groups (eg, patients with frequent ED visits)?	1	1	1	6	Low	SIM
What factors of the payment and policy landscape (eg, mandates and funding) encourage/incentivize or discourage EDs from implementing social risk/need screening?	0	1	2	4	Low	BFS

* *SIM*, screening implementation mechanics (Gap 1); *OCE*, outreach and community engagement (Gap 2); *BFS*, barriers and facilitators to screening (Gap 3); *ED*, emergency department; *EHR*, electronic health record.
